# Encapsulation Processes: Valorization, Stabilization, and Commercialization of Active and Natural Compounds

**DOI:** 10.3390/foods14050844

**Published:** 2025-02-28

**Authors:** Berta Nogueiro Estevinho

**Affiliations:** 1 LEPABE—Laboratory for Process Engineering, Environment, Biotechnology and Energy, Faculty of Engineering, University of Porto, Rua Dr. Roberto Frias, 4200-465 Porto, Portugal; berta@fe.up.pt or berta.estevinho.20@gmail.com; 2 ALiCE—Associate Laboratory in Chemical Engineering, Faculty of Engineering, University of Porto, Rua Dr. Roberto Frias, 4200-465 Porto, Portugal

Encapsulation techniques have emerged as an important strategy for creating nutraceuticals and fortified foods and beverages that contain various bioactive compounds, due to their potential health benefits [1,2,3,4]. Micro and nanoencapsulation are techniques used to create physical barriers between sensitive compounds and external factors [5]. These methods have the potential to improve the stability and efficiency of various ingredients, allowing for the modification of numerous food products (Contribution 1). They contribute to the development of innovative qualities with beneficial effects on human health. Therefore, encapsulation enables the protection and controlled release of several active ingredients [5]. In the literature, numerous microencapsulation techniques are described that can be used to encapsulate flavors, vitamins, stabilizers, probiotics, essentials oils, natural antioxidants, bioactive proteins, and enzymes, among others [6,7,8,9,10,11].

Additionally, compounds extracted from agricultural byproducts within the agri-food sector can be microencapsulated and integrated into various products to enhance their properties [12,13] (Contribution 2). The reuse and valorization of byproducts, alongside the efficient use of raw materials, are key concerns both in the EU and globally [11,14,15]. The incorporation of vegetable byproducts supports a circular economy model by generating eco-friendly and health-beneficial compounds [16,17,18]. The application of a circular economy in agro-industrial activities fosters strategies that not only address environmental challenges, but also enable the creation of value from waste materials [18,19]. Around 89 million tons of food are discarded annually in the European Union. These byproducts still hold potential for use in other economic and industrial sectors due to their unique physical, chemical, and microbiological properties [12,20,21]. As a result, agricultural and food wastes should be seen as valuable resources for producing high-value products, a crucial step toward achieving a zero-waste economy [22,23,24,25].

This Special Issue presents recent research advancements on the encapsulation, development, stabilization, and market application of microencapsulated formulations that incorporate active natural compounds, especially those derived from agricultural byproducts. Specifically, this Special Issue comprises seven research papers that investigated innovative encapsulation techniques and matrices. They also focused on the physicochemical properties of encapsulation systems, examining their impact on food applications, including sensory attributes and nutritional value. The simulated digestion behavior of food products containing encapsulated compounds was also studied by some authors. Furthermore, current strategies to enhance the encapsulation and valorization of specific active and natural compounds were explored.

Two of the papers in this Special Issue focus specifically on the microencapsulation of extracts of byproducts: Otálora et al. (Contribution 3) studied the microencapsulation of betaxanthin pigments from pitahaya (*Hylocereus megalanthus*) byproducts and Couto et al. (Contribution 2) studied the microencapsulation of extracts of wild strawberry (*Fragaria vesca*) byproducts.

Otálora et al. (Contribution 3) found that pitahaya (*Hylocereus* spp.) peel, a byproduct discarded after the consumption or processing of the fresh fruit into juice, accounts for approximately 30% of the fruit’s total weight. This byproduct is a rich, untapped source of bioactive compounds, including polyphenols, betalains (natural pigments such as betacyanins, red-purple substances, and betaxanthins, yellow-orange substances), lycopene, flavonoids, phytosterols, carotenoids, dietary fiber, and pectin [26,27]. These compounds can be utilized by the food industry as functional natural ingredients, namely, in the development of new beverages and colorants. In this study, a betaxanthin-rich extract from pitahaya peel was microencapsulated through spray drying, using pitahaya peel mucilage and maltodextrin as wall materials (Contribution 3). Spray-drying is an efficient method for improving the stability of bioactive substances by protecting them from oxidation and degradation, while also producing powdered formulations. These powders are more convenient and safer to transport, store, and process than liquid formulations [28]. Both types of microencapsulates retained high betaxanthin content and antioxidant activity. The powdered microencapsulates were incorporated into the formulation of candy gummies, which served as a food model and were subjected to in vitro gastrointestinal digestion. These authors concluded that the microencapsulation of betaxanthins with pitahaya peel mucilage could be used as a natural food colorant, replacing synthetic alternatives. This approach may help develop products with health benefits, meeting the increasing consumer demand for healthier products (Contribution 3).

Couto et al. (Contribution 2) highlighted the valorization of agricultural byproducts from wild strawberry (*Fragaria vesca*) through the production of value-added micro/nanostructures using electrohydrodynamic techniques. *Fragaria* vesca is a member of the Rosacea family and is usually found in Europe, Asia (west of the Urals), and North America [29,30,31,32]. This plant has a rich history in traditional medicine, where various beneficial biological effects of strawberry fruit consumption have been documented [29,30,31,33]. These beneficial effects have been mostly attributed to phenolic compounds [34,35]. In the agricultural process, the leaves of F. vesca are usually thrown away and treated as waste [32]. Nevertheless, they can be a rich source of valuable, biologically active substances with a wide range of applications. Couto et al. chose electrohydrodynamic (EHD) techniques for encapsulation due to their inherent advantages, such as the absence of high temperatures and extreme pH conditions, unlike traditional encapsulation methods [36] (Contribution 4). EHD processes use high-voltage electrostatic fields to electrically charge the surface of a polymer solution jet [36] (Contribution 4). Depending on the operational conditions, various microstructures can be obtained, including micro/nanoparticles, fibers, and films [36] (Contribution 4). In this study, different types of zein micro/nanostructures incorporating extracts from *Fragaria vesca* byproducts or quercetin (one of the main polyphenols in the plant) were produced and analyzed using numerous techniques. The main release mechanism identified was “Fickian Diffusion”. However, the authors reported that some antioxidant activity was lost during the encapsulation process or storage of the samples. Ultimately, the micro/nanostructures achieved polyphenol supplementation ranging from 10 to 50 mg of polyphenols per gram of powder. In conclusion, the electrohydrodynamic technique effectively encapsulated the *Fragaria vesca* byproduct extract and quercetin with zein, resulting in micro/nanostructures with different morphologies. These encapsulated structures show potential for use in various fields, including food and nutraceutical applications.

Four other papers in this Special Issue focus on the microencapsulation of extracts of plants: *Laurus nobilis* L. leaf extract (Contribution 5), Perilla skin extract (Contribution 6), *Tecoma stans* extracts (Contribution 1), and indicaxanthin-rich *Opuntia Green* extracts (Contribution 7).

Parralejo-Sanz et al. (Contribution 7) investigated the encapsulation of indicaxanthin-rich *Opuntia* green extracts using double emulsions to enhance their stability and bioaccessibility. *Opuntia ficus-indica* var. *Colorada* fruit is a significant source of indicaxanthin, a betalain known for its antioxidant, anti-inflammatory, and neuromodulatory properties, which have been demonstrated in both in vitro and in vivo models. Indicaxanthin is also considered a natural colorant. Parralejo-Sanz et al. (Contribution 7) prepared double emulsion systems (W1/O/W2) using Tween 20 (TW) and sodium caseinate (SC) as surfactants to encapsulate Colorada fruit pulp extracts. High encapsulation efficiencies were obtained. An evaluation of the in vitro gastrointestinal stability and bioaccessibility was carried out using the standardized INFOGEST© protocol. The double emulsion prepared with Tween 20 showed bioaccessibility values of up to 82.8 ± 1.5% for the main bioactives (indicaxanthin, piscidic acid, and isorhamnetin glucoxyl-rhamnosyl-pentoside 2 (IG2)), highlighting its promising potential as a functional natural colorant.

García-Jiménez et al. (Contribution 1) studied the microencapsulation of *Tecoma stans* extracts using a spray drying technique and performed an analysis of bioactive property preservation and physical characterization. Maltodextrin (MD), arabic gum (AG), and a 1:1 blend (MD/AG) were used as encapsulating agents. Spray drying decreased the antioxidant activity of the extract. The encapsulated infusion with MD/AG exhibited the highest hypoglycemic activity, as indicated by its low glycemic index (GI = 47). Based on the results, the microencapsulates have the potential to be incorporated into food products to enhance nutritional quality and help prevent or treat various health conditions (Contribution 1).

The microencapsulation of *Perilla* skin extract was studied by Kang et al. (Contribution 6). The authors explored the formulation and evaluation of a sustainable oil-in-water emulsion. The emulsion incorporates *Perilla* skin extract, which is rich in antioxidants, and upcycled aquasoya powder, a byproduct derived from soy protein. The focus of the study was on evaluating the physical stability and antioxidant properties of this emulsion (Contribution 6). This study used aquasoya powder, a promising emulsifier, to incorporate antioxidant compounds from Perilla skin extract (PSE) into an oil-in-water emulsion. The results showed that the emulsion with PSE had a smaller droplet size and improved physical stability compared to the emulsion without PSE, maintaining stability over 30 days. The emulsion with PSE also demonstrated higher antioxidant activity and slower lipid oxidation, indicating superior antioxidant properties. Therefore, it was proven that aquasoya is an effective emulsifier for stabilizing antioxidant compounds from perilla skin in O/W emulsions (Contribution 6).

The encapsulation of *Laurus nobilis* L. leaf extract using an electrostatic extrusion process was studied by Dobroslavić et al. (Contribution 5). Bay leaves (*L. nobilis* L.) are rich in polyphenols, which have potential for functional food applications but face challenges in stability and bioaccessibility. These issues can be addressed by using microencapsulation techniques, such as electrostatic extrusion. This study evaluated the use of electrostatic extrusion for encapsulating bay leaf polyphenol (BLP), focusing on the evaluation of the polyphenolic content, antioxidant activity, release kinetics, and bioaccessibility (Contribution 5). The results showed that using 1% alginate, 1.5% CaCl_2_, and 0.5% chitosan achieved the highest encapsulation efficiency (92.76%) and antioxidant activity. The method also enhanced the controlled release and bioaccessibility of BLP, demonstrating that electrostatic extrusion is an effective technique for BLP encapsulation (Contribution 5).

The last paper published in this Special Issue concerns the potential benefits of combining astaxanthin and anthocyanin in nanoparticles, investigating their effects on lipid levels and oxidative stress in *Caenorhabditis elegans* (*C. elegans*) [37]. Currently, there is a global push to use natural bioactive compounds in the development of functional food supplements. These supplements can complement traditional methods for managing obesity, which is associated with a range of serious health problems, including type II diabetes, cardiovascular diseases, reproductive failure, certain cancers, and premature death. This paper explores the effects of astaxanthin–anthocyanin nanoparticles (AXT-ACN NPs) on lipid accumulation and antioxidative capacity in C. elegans subjected to a high-sugar, high-fat diet, simulating obesity. The results showed that AXT-ACN NPs improved the lifespan, motility, and reproductive capacity of high-fat C. elegans [37]. The nanoparticles also reduced fat and lipofuscin accumulation, and the level of reactive oxygen species (ROS). Additionally, AXT-ACN NPs enhanced the activity of antioxidant enzymes (catalase, superoxide dismutase, and glutathione peroxidase) while decreasing malondialdehyde (MDA) levels. The study suggests that AXT-ACN NPs, through their synergistic antioxidant and lipid-lowering effects, could be useful in functional foods targeting obesity-related health issues. Moreover, the microencapsulation of these bioactive compounds appears fundamental to obtaining their health benefits [37].

The papers in this Special Issue highlight ongoing research on the microencapsulation of bioactive compounds for food applications worldwide. Several microencapsulation methodologies were adopted in the studies. A key area for future research is the valorization of byproducts and the extraction of value-added bioactive compounds from them. This approach could lead to more sustainable practices and the discovery of new beneficial compounds for various applications. With impressive results and significant potential for further exploration, microencapsulation is seen as a promising technique for the creation and reformulation of innovative food products.

The editor hopes this Special Issue will inspire readers in the field and encourage the development of new ideas or methodologies for future research.
